# Surgimiento y evolución de los sistemas de normalización para laboratorios clínicos

**DOI:** 10.1515/almed-2024-0064

**Published:** 2024-07-12

**Authors:** Rita B. Khattar, Maha E. Nehme

**Affiliations:** Holy Family University Batroun, Liban-Nord, Líbano; Internal Medicine Physician, Master in Health and Hospital Management, Holy Family University Batroun, Liban-Nord, Líbano

**Keywords:** normalización, laboratorios clínicos, ISO

## Abstract

**Introducción:**

La historia y evolución de la normalización de las actividades de laboratorio clínico va acompasada con el desarrollo de normas de calidad internacionales.

**Contenido:**

En el presente estudio, presentamos los hitos que marcan las diferentes etapas en la evolución de los sistemas de calidad, a lo largo de las últimas décadas. La acreditación del cumplimiento de las normas nacionales e internacionales de calidad es obligatoria en la mayoría de los países. La aplicación de normas de calidad garantiza el reconocimiento internacional de la validez de los resultados informados a multitud de actores, especialmente a pacientes y médicos.

**Resumen:**

La nueva norma ISO 15189 es la norma internacional más específica dirigida a todo tipo de laboratorios clínicos.

**Perspectiva:**

Sería necesario investigar más a fondo si las prácticas de laboratorio reflejan la evolución real de los estándares dentro del campo del laboratorio médico.

## Introduction

Uno de los objetivos del laboratorio clínico es proporcionar orientacion para llegar a un diagnóstico médico. Sin embargo, este diagnóstico se puede retrasar debido a la imprecisión de los resultados, terapias inadecuadas, o solicitudes de pruebas innecesarias, lo que deriva en incremento de costes, pérdida de tiempo y exceso de gasto en personal [1].

A nivel mundial, los laboratorios han reconocido la importancia de operar dentro de un sistema global de gestión de la calidad altamente efectivo que garantice la mejor ejecución de todos los procesos, con el fin de corregir los errores que surgen de estos procesos y evitar sus consecuencias [2, 3]. Para tal fin, se desarrollaron repositorios estandarizados que proporcionaban técnicas para la identificación de no conformidades en cada una de las fases analíticas. Sin embargo, el establecimiento de un sistema sólido de gestión de la calidad “permite tener un laboratoriode alta calidad con capacidad para detectar errores y prevenir su recurrencia, aunque no garantiza la ausencia total de errores en un laboratorio” [1].

Los profesionales sanitarios son cada vez más conscientes de los beneficios que aporta contar con un sistema eficaz de gestión de la calidad, entre los que se encuentran una mayor trazabilidad y calidad de los resultados [2, 3], una reducción en el número de errores analíticos [1], menores costes derivados de la mala calidad de los resultados [4, 5], la estandarización de las técnicas, y la cooperación entre todos los miembros del equipo a la hora de implementar el sistema de calidad.

Por lo tanto, la estandarización efectiva promueve la percepción de valores por parte de los miembros del equipo, además de mejorar la comunicación interna y el empoderamiento de los empleados [2, 6].

El objetivo de esta minirevisión es ofrecer una visión general del desarrollo y evolución de las normas aplicadas por los sistemas de gestión de la calidad en los laboratorios clínicos.

## Normalización: definición y tipología

“Una norma (o estándar) permite establecer directrices, así como un procedimiento para las operaciones realizadas en diferentes sectores”. Un estándar determina el proceso óptimo a adoptar para las diferentes actividades de una empresa, con el fin de combinar eficiencia, seguridad y fiabilidad [7].

La estandarización puede ser llevada a cabo a nivel internacional, nacional o local, siendo la Organización Internacional de Normalización (*International Organization for Standardization*, ISO) el principal organismo mundial de estandarización. El *National Committee for Clinical Laboratory Standards* (NCCLS), actualmente llamado Instituto de Normas Clínicas y de Laboratorio (*Clinical and Laboratory Standards Institute*, CLSI), desarrolla normas y políticas de utilidad para el ámbito de la medicina. Otros ejemplos son la Organización Mundial de la Salud (OMS) y el Comité Europeo de Normalización (CEN).

Sin embargo, los estándares internacionales pueden ser difíciles de adaptar a un país dado debido a su falta de especificidad. Así, los estándares nacionales pueden desarrollarse en base a estándares internacionales y personalizarse según los requisitos del país en cuestión [1].

El *American National Standards Institute* (ANSI), la *Standardization Administration of China* (SAC), el *Deutsches Institut für Normung* (DIN), la *British Standards Institution* (BSI), el *Japanese Industrial Standards Committee* (JISC) y la *Canadian Standards Association* (CSA) son los equivalentes nacionales de la Agencia Francesa de Normalización (*Association Française de Normalisation*, AFNOR), que consulta oficialmente cada proyecto de norma francesa, europea o internacional en Francia [8].

## Aparición y evolución de los sistemas de estandarización en los laboratorios clínicos

Estados Unidos (EEUU) fue el primer país en mostrar interés por la calidad en los laboratorios clínicos. Ya en 1967, se redactó la Ley de Mejora de los Laboratorios Clínicos (*Clinical Laboratory Improvement Act*, CLIA) (81 Stat. 536, Public Law 90–174, Section 5) para establecer estándaresde calidad de los análisis clínicos en muestras humanas [9].

Además, el NCCLS, creado oficialmente en 1968, publicó en 1969 la guía de normas de calidad titulada “Preparación de Manuales para la Instalación, Uso y Reparación de Instrumentos de Laboratorio” (*“Preparation of Manuals for Installation, Operation and Repair of Laboratory Instruments”)*. A lo largo de la década siguiente, se crearon veinte estándares de laboratorio clínico. El NCCLS empezó a ganar reconocimiento a nivel interacional en 1985, y la OMS lo nombró uno de susCentros Colaboradores para el Desarrollo de Estándares de Laboratorio Clínico. Así mismo, con el fin de abarcar todas las áreas de laboratorio clínico, los tres comités básicos del NCCLS (química clínica, hematología y microbiología) se han ampliado a ocho [10].

En 1988, se declaró la CLIA’88 para reemplazar la CLIA’67 mediante Enmienda de la Ley de Servicios de Salud Pública (*Public Health Services Act Amendment*) (102 Stat. 2903, Public Law 100–578) [9]. Implementada en febrero de 1992 [11], su objetivo era mejorar todos los laboratorios clínicos de EEUU y garantizar la calidad de sus pruebas, dondequiera que se realizaran [1]. Este programa aún se sigue utilizando a día de hoy bajo el mismo nombre CLIA’88, aunque posteriormente se realizaron dos revisiones, una en 1997 y otra en 2012 [9, 11].

De hecho, el Colegio de Patólogos Americanos (CAP) utiliza la CLIA como parte del Programa de Acreditación de Laboratorios (*Laboratory Accreditation Program*, LAP) para la acreditación de laboratorios clínicos en todo el mundo, dándole un aspecto internacional a pesar de ser una regulaciónnacional muy específica de EEUU para respaldarlas prácticas de calidad. Excepto paraensayos clínicos e investigación básica, este documento es aplicable a todas las pruebas de laboratorio clínico realizadas en humanos y abarca disciplinas específicas, como la histopatología o las pruebas genéticas.

El programa CLIA se centra principalmente en la gestión y el control de procesos, más que en clientes y personal [12]. Este programa contiene varias subpartes que tratan, entre otras cosas, disposiciones generales (Subparte A), estándares y condiciones para las pruebas de competencia por especialidad y subespecialidad (Subparte H), así como un sistema general de calidad de laboratorio que abarca las fases preanalíticas, analítica y postanalítica, detallado también por especialidad y subespecialidad (Subparte K) [13].

Con dichas directrices y estándares, se ha logrado mejorar la calidad de las pruebas analíticas. Sin embargo, no es hasta los años noventa cuando se empieza a mostrar verdadero interés en la gestión de inventario en el laboratorio. La NCCLS publicó en 1994 el documento GP6-A “Sistemas de Control de Inventario para Suministros de Laboratorio; Directrices Aprobadas” (“*Inventory Control Systems for Laboratory Supplies; Approved Guidelines”*). En dicho documento, se establecieron las directrices para los sistemas de control de inventario, destinadas a garantizar la disponibilidad de reactivos y suministros en el laboratorio [14].

De manera similar, la *Joint Commission International* (JCI) acredita, según sus propios estándares, a organizaciones sanitarias en más de 100 países [15]. En dicho contexto, ha desarrollado estándares y directrices para los laboratorios hospitalarios [12].

Francia fue el primer país en establecer una norma de calidad para los laboratorios clínicos, la llamada *“Guide de Bonne Execution des Analyses Médicales”* (GBEA, “Guía de buenas prácticas en los análisis clínicos”). En el decreto inicial (GBEA I), publicado en París el 2 de noviembre de 1994, se establecían las directrices que debían aplicar los laboratorios clínicos.

En contraposición con las normas americanas, la GBEA estaba orientada a promover una metodología de trabajo que garantizara la obtención de resultados precisos y exactos, más que a establecer un procedimiento contreto para cada tipo de prueba analítica. En esta metodología, se incluían todos los pasos para la obtención, procesamiento y comunicación de resultados de muestras biológicas [16]. La GBEA I exigía que todos los procedimientos y técnicas analíticas quedaran registrados por escrito [17]. Esto último, relacionado con el control de calidad, era una parte esencial del sistema de garantía de la calidad en los laboratorios clínicos [16].

El segundo decreto (GBEA II) constituyó una revisión de laGBEA I, con fecha del 26 de noviembre de 1999 y publicado en el Boletín Oficial el 11 de diciembre de 1999 [18]. Así mismo, cuenta con un anexo titulado “Guía para el buen uso de los equipos informáticos” *(“Guide to the Good Use of Computers, GBUI”) ()* [17]. La GBEA aborda aspectos sanitarios en general, y la calidad de la biología clínica en Francia en particular. Además, hace hincapie en el importante papel que desempeñan los biólogos, especialmente en las actividades de las fases preanalítica y postanalítica [18].

Posteriormente, la ISO, cuyo comité técnico 212 (ISO/TC 212) fue organizado por el NCCLS [10], estableció en 1999 la norma ISO 17025 “Requisitos generales para la competencia de los laboratorios de ensayo y de calibración” (*“General requirements concerning the competence of laboratories for calibration and testing’”*) [19]. Dicha norma es una revisión de “Requisitos generales para la competencia de los laboratorios de ensayo y calibración” (“*General requirements for the competence of calibration and testing laboratories*”) [20]. Sin embargo, estas normas no determinan el grado de cumplimiento de las recomendaciones legales y de seguridad por parte de los laboratorios [19].

Por otro lado, en 2002, el NCCLS publicó una guía llamada GP2-A4 “Manuales de Procedimientos Técnicos en el Laboratorio Clínico; Guía Aprobada”, 4^a^ edición (*“GP2-A4 Clinical Laboratory Technical Procedure Manuals; Approved Guideline – Fourth Edition”*), que contiene instrucciones para el desarrollo, revisión, aprobación, gestión y uso de políticas, procesos y procedimientos de análisis clínicos [14].

Después, en febrero de 2003, se publicó la primera edición de la norma ISO 15189:2003, “Laboratorios Clínicos. Requisitos Particulares Relativos a la Calidad y la Competencia” (“*Laboratories for Medical Analysis-Specific Requirements Concerning Quality and Competence*”), rectificada en julio de 2003 [21]. Estas normas son los primeros requisitos internacionales dirigidos específicamente a los laboratorios clínicos y, a diferencia de la norma ISO 17025, que únicamente aborda el control de los procedimientos técnicos de cada prueba analítica, abarca todos los ámbitos de aplicación [1, 22]. Las recomendaciones de la 1^a^ edición de la ISO/TR 22869:2005 se publicaron en febrero de 2005 para facilitar la implementación de la norma 15,189:2003 en los laboratorios europeos [23].

En 2004, a raíz de la publicación de esta norma, en Francia se modificó el GBEA. Dichos cambios afectaban principalmente a la metrología, los sistemas informáticos, el transporte de muestras biológicas, la biología molecular y la inmunohematología [24]. A pesar de todas estas modificaciones, la mera implementación del GBEA no garantiza un sistema adecuado de garantía de calidad [18]. De hecho, el GBEA regula un sistema de garantía de calidad que, a diferencia de la norma ISO 15189, no exige la presentación de un manual de garantía de calidad, ni cubre una serie de preocupaciones, como las responsabilidades del personal, la revisión de contratos, la subcontrata de laboratorios, la mejora continua, o la revisión de la gestión, entre otros [24].

El GBEA ayuda a los laboratorios clínicos a organizarse durante el proceso de acreditación de la *Agence Nationale d’Accréditation et d’Évaluation en Santé* (Agencia Nacional Francesa de Acreditación y Evaluación Sanitarias, ANAES) [18].

El control de la seguridad de los procedimientos analíticos también se reconoce en EEUU. El NCCLS publicó en 2004:“HS1-A2 Modelo de sistema de gestión de la calidad para la atención sanitaria” (“HS1-A2 *A Quality Management System Model for Health Care; Approved Guideline-Second Edition”)*; “GP17-A2 Seguridad en el laboratorio clínico” *(“GP17-A2 Clinical Laboratory Safety; Approved Guideline-Second Edition”)* para la implantación de un programa de seguridad adaptable a cualquier laboratorio; y “GP26-A3 Aplicación de modelo de seistema de gestión de la calidad para los servicios de laboratorio” *(“GP26-A3 Application of a Quality Management System Model for Laboratory Services; Approved Guideline-Third Edition”)*, que describe la secuencia de actividades en el laboratorio clínico y proporciona la información necesaria para mejorar los procesos y cumplir así la legislación nacional y los requisitos de acreditación [14].

A su vez, en mayo de 2005 se publicó una actualización de la norma ISO 17025:1999 [25], así como una corrección en 2006 [26]. Las recomendaciones de la norma ISO/IEC 17025 emitida en 1999 pasaron a ser requisitos en 2005 [27]. Dado que las norma ISO 17025 e ISO 9001 poseen unas estructuras extremadamente similares, y dado que su versión de 2005 fue desarrollada de manera que fuera compatible con la edición del año 2000 de las normas de la serie ISO 9000, los laboratorios que la cumplen suelen funcionar de acuerdo a dichos principios [28, 29]. Posteriormente, en abril de 2007 se publicó la 2^a^ edición de la norma ISO 15189:2007 “Laboratorios clínicos. Requisitos particulares para la calidad y la competencia”, que fue revisada en septiembre de 2007 [30].

En abril y septiembre de 2007, se publicaron y modificaron las normas de la 2^a^ edición de la norma ISO 15189:2007 *“Laboratorios clínicos. Requisitos particulares para la calidad y la competencia”* [30]. “Esta norma internacional, que está basada en las normas ISO/IEC 17025 e ISO 9001, establece las competencias y los estándares de calidad para los laboratorios que realizan análisis clínicos. Se reconoce que un país puede tener sus propias regulaciones o requisitos particulares que pueden aplicarse a toda o parte de la profesión y sus actividades y obligaciones relacionadas” [31]. La cuarta edición del “Modelo de sistema de gestión de la calidad para el laboratorio clínico” *(“Quality Management System: A Model for Laboratory Services”),* también llamado GP26-A4 [10], fue lanzada en 2011 por el NCCLS, ya conocido como CLSI. Las dos directivas anteriores, HS01-A2 y GP26-A3, están contempladas de manera conjunta en esta nueva versión [32]. Tanto la norma ISO 15189 como las directrices del CLSI se centran en los controles de calidad que deben realizarse a lo largo de todo el proceso analítico de un laboratorio (en las tres fases: preanalítica, analítica y postanalítica) para obtener un producto o servicio de calidad [33].

En contraposición a la norma ISO 15189, que básicamente enumera los requisitos sin aportar aclaración adicional alguna, el documento de recomendaciones GP26 del CLSI ofrece una descripción considerablemente más detallada de la integración de la gestión de la calidad en las distintas actividades del laboratorio clínico. En dicho documento también se abordan las subdisciplinas de laboratorio, ofreciendo recomendaciones específicas para cada una de ellas. Por todas estas razones, estas directivas se aplican a nivel internacional a la hora de implementar los requisitos de la norma ISO 15189 [12].

Un modelo basado en doce componentes clave del sistema de calidad (QSE) – organización, orientación al cliente, instalaciones y seguridad, personal, gestión de suministros e inventario, equipos, gestión de procesos, documentos y registros, gestión de la información, gestión de eventos no conformes (NCE) o problemas, evaluaciones y mejora continua – proporciona un marco para la gestión de procesos del laboratorio. En 2011 se publicó una revisión de la directiva GP26-A4, que describe un sistema integrado de QSE y de rutas de flujo de trabajo. Dicha edición ha sido reorganizada para enfatizar la importancia de las responsabilidades de liderazgo, la reducción de errores y la mejora de la eficacia y eficiencia de las actividades de laboratorio. Estos elementos sustentan las rutas de flujo de trabajo y las distintas disciplinas de laboratorio [32].

En septiembre de 2011, la JCI confirmó que suscribía los elementos clave del QSE del CLSI y/o la mayoría de los requisitos específicos de la versión de 2007 de la norma ISO 15189, así como el seguimiento de la evolución de sus actualizaciones. Al igual que ISO, las normas del JCI van más allá de los requisitos mínimos de la CLIA [34]. Las Normas y Directrices de la JCI organizan las actividades de los diferentes sectores de laboratorio, con el objetivo de proporcionar normas de calidad para cada sector o especialidad [12].

Las normas ISO se revisan y actualizan cada cinco años. La 3^a^ edición de la norma ISO 15189 “Laboratorios clínicos. Requisitos particulares para la calidad y la competencia” se actualizó en 2012, habiendo sido publicada en noviembre del mismo año [35].

Como parte de los diversos cambios introducidos en su edición de 2007, la revisión de 2012 de la norma especifica el enfoque por procesos, aborda la gestión de riesgos y uniformiza la gestión de la información del laboratorio [36]. Además de monitorear consistentementela satisfacción y fidelidad de pacientes y médicos, define criterios para la documentación, impone normas para las guías de automatización y exige la elaboración de un programa de bienvenida para nuevos empleados [37].

De este modo, a fecha del 1 de enero de 2017, entró en vigor la 3^a^ versión de las normas del JCI para la acreditación de laboratorios clínicos. Esta versión contiene secciones sobre normas centradas en el paciente, incluyendo así mismo normas para la gestión de organizaciones sanitarias, que abarcan la gobernanza, el liderazgo y la dirección, la gestión de la información, las credenciales y la cualificación del personal, las instalaciones y la seguridad, así como el método para el proceso de control de calidad, que tiene en cuenta tanto las áreas comunes como las específicas, como el control de calidad de las especialidades, las pruebas analíticas por especialidades, así como el banco de sangre y las transfusiones [38].

En noviembre de 2017 [27], se publicó una nueva versión de las normas ISO/IEC 17025:2005/Cor1:2006, con un mejor enfoqueen la gestión, la evaluación y la prevención de riesgos, así como en el enfoque por procesos, las notificaciones del sistema relacionadas con el sistema de gestión, las reglas de toma de decisiones, la imparcialidad, la competencia y la coherencia de las operaciones de laboratorio. Dichos criterios son aplicables a todas las instituciones que realizan actividades de laboratorio [28]. Esta nueva versión incluye dos anexos, uno que detalla el potencial para integrar un sistema de gestión ISO 9001, y otro sobre la trazabilidad metrológica [39].

Los laboratorios a los que se dirige esta versión son los que realizan calibraciones, pruebas y muestreos; aquellos que sirven como primera, segunda o tercera parte; aquellos que realizan operaciones de inspección o ayudan en el proceso de certificación; aquellos que tienen instalaciones permanentes o sistemas móviles; y aquellos que forman parte de una organización [17].

Aunque los requisitos técnicos para los sistemas de gestión de calidad dentro del estándar ISO/IEC 17025 son comparables a los de ISO/IEC 15189, el paciente y el médico que prescribe no se mencionan en el estándar ISO/IEC 17025.

Posteriormente, el 1 de enero de 2022, se publicó la 4^a^ edición de las normas de la JCI para la acreditación de laboratorios clínicos [40].

Así mismo, tras las revisiones propuestas y publicadas el 4 de febrero de 2019, el 11 de julio de 2022 se publicó una nueva norma CLIA en el boletín oficial *Federal Register* (87 FR 41194) titulada “Enmiendas a la Ley de Mejora de los Laboratorios Clínicos de 1988 (CLIA)-Reglamentos sobre pruebas de aptitud relacionadas con los analitos y la calidad aceptable” *(*“*Clinical Laboratory Improvement Amendments of 1988 (CLIA) Proficiency Testing Regulations Related to Analytes and Acceptable Performance”)*. Esta nueva regla fue implementada parcialmente el 10 de agosto de 2022 y se aplicará a partir del 11 de julio de 2024 para el resto de las modificaciones [11].

Diez años después, la norma ISO 15189:2012 ha sido revisada, habiendo sido publicada la 4^a^ edición de *“Laboratorios clínicos. Requisitos particulares para la calidad y la competencia”* en diciembre de 2022. Esta última es de aplicación en el ámbito de los laboratorios de biología médica y otros tipos de laboratorios clínicos, como los de anatomía patológica y de citología [41].

También se han desarrollado varias normas ISO en las que se establecen requisitos aplicables a las actividades de los laboratorios clínicos. Por ejemplo, la norma ISO 13485:2016 “Productos sanitarios. Sistemas de gestión de la calidad. Requisitos para fines reglamentarios” [42]; la norma EN ISO 10012 “Sistemas de gestión de las mediciones. Requisitos para los procesos de medición y los equipos de medición” que aborda “la gestión de los instrumentos y procesos analíticos” [22]; y la norma ISO 22870:2016 “Análisis junto al paciente (*Point of Care Testing*, POCT). Requisitos de calidad y competencia”, que establece las condiciones para los estudios de biología médica externalizados y que todas las instituciones sanitarias que ofrezcan “atención ambulatoria” deben aplicar paralelamente a la norma ISO 15189 [43].

La edición inicial de la norma ISO 22870 se publicó en 2006 y se actualizó en 2016 con una revisión menor relacionada con la versión más reciente, la ISO 15189:2012.

La norma ISO 22870 es de aplicación cuando las pruebas analíticas junto al paciente (POCT) se realizan “en un hospital, clínica o institución sanitaria que proporciona asistencia ambulatoria” para “mediciones transcutáneas, análisis de aire expirado y monitorizaciòn *in vivo* de parámetros fisiológicos”.

No obstante, esta directriz no incluye el autodiagnóstico realizado por los pacientes en casa o en una clínica [43]. Los quirófanos, servicios de urgencias y unidades de cuidados intensivos son sólo algunos de los servicios hospitalarios que están cubiertos por el POCT, que incluye la medición de la CPK, la mioglobina y la troponina [44], además de los gases sanguíneos deslocalizados [45].

Este sistema de gestión de la calidad incluye la evaluación y aprobación de propuestas y protocolos de usuarios finales, la compra e instalación de equipos, “el mantenimiento de consumibles y reactivos; la formación, acreditación y reacreditación de usuarios de sistemas POCT, y el control y garantía de calidad”. Así mismo, aborda la gestión de riesgos para el paciente y el sistema sanitario [43].

Las directrices de esta última norma ISO 22870 han sido incorporadas recientemente a la norma ISO 15189:2022, habiendo sido retirada la versión ISO 22870:2016 [41].

Actualmente, existen normas específicas para actividades de laboratorio, como la norma CAN/CSA-Z902 “Sangre y productos sanguíneos” *(“Blood and blood products”),* redactada por el Grupo CSA para organizaciones que “cuentan con un laboratorio de banco de sangre, un programa de donación autóloga o un programa de donante ambulatorio”, y se crean específicamente enfocadas a sectores o ámbitos académicos concretos [1, 46]. Entre estas normas, se incluyen la norma ISO 20776-1:2019 para el diagnóstico *in vitro* de susceptibilidad antimicrobiana y la evaluación del rendimiento de dispositivos para antibiogramas – Parte 1: método de referencia de microdilución en caldo para evaluar la susceptibilidad *in vitro* a agentes antimicrobianos de bacterias aerobias de crecimiento rápido implicadas en enfermedades infecciosas [47] o la norma H15: “Referencia y Procedimientos Seleccionados para la Determinación Cuantitativa de la Hemoglobina en Sangre, Tercera Edición” *(“Reference and Selected Procedures for the Quantitative Determination of Hemoglobin in Blood, Third Edition”)* [48].

## Aplicación de la norma ISO 15189

La norma ISO 15189 es actualmente aceptada como referencia fundamental para la acreditación de laboratorios clínicos [41]. La Federación Europea de Química Clínica y Medicina de Laboratorio (EFLM) y la Federación Internacional de Química Clínica y Medicina de Laboratorio (IFCC) admiten que la norma ISO 15189 es la norma de referencia para los laboratorios. Así, “la EFLM y la IFCC reconocen que la norma ISO 15189 especifica con exactitud los requisitos de calidad y competencia de los laboratorios clínicos” [3].

Aquellos países que optan por aplicar la norma ISO 15189 para verificar la aptitud de los laboratorios adoptan un procedimeinto normalizado [49]. Además, dado que su aplicación es opcional, los gobiernos u otras autoridades puede recomendar la adhesión a la norma [1]. Sin embargo, en los países en los que no es obligatoria la acreditación, los laboratorios pueden ser acreditados por un organismo de otro país [49].

Para la acreditación de los laboratorios clínicos, solo el 59 % de los organismos de acreditación de los países europeos adoptan la norma ISO 15189 como referencia [50, 51]. En EEUU, antes de solicitar la acreditación ISO 15189 con el CAP 15189, el laboratorio debe obtener primero la acreditación del CAP-LAP, que se basa en la ley CLIA, de cumplimiento obligatorio en EEUU. De hecho, la norma ISO 15189 no responde a los requisitos de la CLIA, por lo que la certificación ISO 15189 no puede sustituir a la certificación CLIA [6]. Por otro lado, la norma ISO 15189 debe mantener cierto nivel de generalidad, para poder adaptarse a las normas de buenas prácticas de los diferentes países, normalmente estructuradas según la disponibilidad de recursos [12].

Cabe señalar que los laboratorios con acreditación CAP-LAP pueden cumplir fácilmente muchos de los requisitos técnicos de la norma ISO 15189, lo que no ocurre con los requisitos de gestión, más difíciles de alinear. Sin embargo, dado que la aplicación de los requisitos de gestión es, sin ninguna duda, eficaz a la hora de mejorar los servicios [6], la norma ISO 15189 es de aplicación universal para los laboratorios de EEUU, lo que los hace potencialmente más competitivos [33]. Si bien en otros países, como los de la Unión Europea, las normas ISO sirven de modelo a los gobiernos, no ocurre lo mismo en EEUU [33], donde el sistema sanitario se basa mayoritariamente en proveedores de seguros médicos privados [52]. Por ello, el CAP acredita a más laboratorios de otros países con sistemas públicos de salud, que suelen requerir la acreditación ISO [33].

Así, el CAP “acredita a más de 8.000 laboratorios en todos sus programas de acreditación, incluidos aproximadamente 437 laboratorios internacionales en más de 50 países” [6].

## Conclusiones

El presente estudio sobre las normas aplicables a los laboratorios clínicos, y su evolución desde la década de los sesenta, demuestra que la normalización es crucial y predice las tendencias de acreditación en un futuro próximo.

La nueva norma ISO 15189 es la norma internacional más específica dirigida a todo tipo de laboratorios clínicos. Este estándar constituye actualmente un soporte básico para la mayoría de las normas relacionadas con el mismo campo.

Los resultados de utilizar este enfoque muestran ventajas significativas tanto para los laboratorios como para los pacientes. Dado que la mayoría de los procedimientos terapéuticos se basan en la información aportada por el laboratorio clínico, sus ventajas en términos de gasto sanitario resultan incuestionables.
